# Involvement of CD40-CD40L and ICOS-ICOSL in the development of chronic rhinosinusitis by targeting eosinophils

**DOI:** 10.3389/fimmu.2023.1171308

**Published:** 2023-06-01

**Authors:** Aina Zhou, Chenxi Shi, Yuhui Fan, Yushuang Zheng, Jue Wang, Zhichen Liu, Huanxia Xie, Jisheng Liu, Qingqing Jiao

**Affiliations:** ^1^ Department of Ear, Nose, and Throat, The First Affiliated Hospital of Soochow University, Suzhou, China; ^2^ Department of Pathology, The First Affiliated Hospital of Soochow University, Suzhou, China; ^3^ Department of Dermatology, The First Affiliated Hospital of Soochow University, Suzhou, China

**Keywords:** CD40-CD40L, ICOS-ICOSL, chronic rhinosinusitis, eosinophils, TNF-α, IL-5, p38 MAPK

## Abstract

**Background:**

Chronic rhinosinusitis (CRS), whose prevalence and pathogenesis are age-related, is characterized by nasal tissue eosinophil infiltration. CD40-CD40 ligand (CD40L) pathway involves in the eosinophil-mediated inflammation, and inducible co-stimulator (ICOS)–ICOS ligand (ICOSL) signal can strengthen CD40-CD40L interaction. Whether CD40-CD40L and ICOS-ICOSL have a role in the development of CRS remains unknown.

**Objectives:**

The aim of this study is to investigate the association of CD40-CD40L and ICOS-ICOSL expression with CRS and underlying mechanisms.

**Methods:**

Immunohistology detected the expression of CD40, CD40L, ICOS, and ICOSL. Immunofluorescence was performed to evaluate the co-localizations of CD40 or ICOSL with eosinophils. Correlations between CD40-CD40L and ICOS-ICOSL as well as clinical parameters were analyzed. Flow cytometry was used to explore the activation of eosinophils by CD69 expression and the CD40 and ICOSL expression on eosinophils.

**Results:**

Compared with the non-eCRS subset, ECRS (eosinophilic CRS) subset showed significantly increased CD40, ICOS, and ICOSL expression. The CD40, CD40L, ICOS, and ICOSL expressions were all positively correlated with eosinophil infiltration in nasal tissues. CD40 and ICOSL were mainly expressed on eosinophils. ICOS expression was significantly correlated with the expression of CD40-CD40L, whereas ICOSL expression was correlated with CD40 expression. ICOS-ICOSL expression positively correlated with blood eosinophils count and disease severity. rhCD40L and rhICOS significantly enhanced the activation of eosinophils from patients with ECRS. Tumor necrosis factor–α (TNF-α) and interleukin-5 (IL-5) obviously upregulated CD40 expression on eosinophils, which was significantly inhibited by the p38 mitogen-activated protein kinase (MAPK) inhibitor.

**Conclusions:**

Increased CD40-CD40L and ICOS-ICOSL expressions in nasal tissues are linked to eosinophils infiltration and disease severity of CRS. CD40-CD40L and ICOS-ICOSL signals enhance eosinophils activation of ECRS. TNF-α and IL-5 regulate eosinophils function by increasing CD40 expression partly *via* p38 MAPK activation in patients with CRS.

## Introduction

Chronic rhinosinusitis (CRS) is a chronic inflammatory disease in the nose and paranasal sinus characterized histologically by the infiltration of inflammatory cells, especially eosinophils, with high prevalence worldwide (1–3). CRS exhibits high heterogeneity due to its numerous etiologies, and age may be one factor. Existing research studies indicate that there is increasing prevalence among elderly patients compared with their younger counterparts as well as higher incidence of nasal polyposis and worse computed tomography (CT) score (4–7). Another factor to consider is the different endotypes of CRS. On the basis of the extent of tissue eosinophilia, CRS can be classified into eosinophilic CRS (ECRS) and non-eosinophilic (non-eCRS) subtypes (8, 9). Compared with non-eCRS, ECRS is associated with worse disease severity, a higher risk of comorbid asthma, and a higher ratio of recurrence and revision surgery (10–12). There are significant geographic and ethnic differences in the tissue eosinophilic infiltration, ECRS is predominant in Western white patients and less common in East Asians (13–15). However, it has been reported that the proportion of ECRS has increased over time in Korea and China (16, 17). Thus, identifying specific mediators that drive the development of eosinophils and modulating their functions, particularly of adult patients with ECRS, will be important for developing novel treatment strategies and improving treatment outcomes.

CD40 is a cell surface receptor that belongs to the tumor necrosis factor–R (TNF-R) family (18). Although the primary function was initially restricted to B and T lymphocytes, CD40 has been explored more extensively because of its broad expression on non-lymphocytic cell types (19–23). Ohkawara et al. reported that eosinophils isolated from allergic subjects express CD40, which is biologically functional. Interestingly, they also found that CD40 was detected in nasal polyp (NP) tissues but not in normal nasal mucosa (inferior turbinate) and primarily in eosinophils. At the same time, they demonstrated that CD40 expression in eosinophils could be upregulated by exposure to immunoglobulin A (IgA) immune complexes and downregulated by interleukin-10 (IL-10) and the synthetic steroid budesonide (24). These observations suggest that the CD40-CD40 ligand (CD40L) pathway may contribute to the development of eosinophil-mediated inflammation. It is therefore reasonable to speculate that the CD40-CD40L signal pathway may be involved in the regulation of eosinophils function in CRS.

CRS without NPs (CRSsNP) and CRS with NPs (CRSwNP) are the two phenotypes of CRS according to the presence or absence of NP (1, 3). CRSwNP is often characterized by the local production of polyclonal IgE idiotypes (25–29). As for the induction and regulation of IgE synthesis, a two-signal model is accepted. The first signal is provided by cytokines IL-4 or IL-13, which are secreted by T cells, mast cells, and basophils. The second signal is CD40-CD40L interaction, which is well established as a key signal for the induction of isotype switching in B cells (30–33). Interestingly, inducible co-stimulator (ICOS)–ICOS ligand (ICOSL) ligation can promote the expression of CD40L, which, in turn, strengthens CD40-CD40L interaction to provide a co-stimulatory signal for B-cell activation. In addition, one very recent study has shown that ICOS co-stimulation induces CD40L expression by human T cells (34, 35). Nevertheless, the role of ICOS-ICOSL and its interaction with CD40-C40L in CRS has not been investigated.

Therefore, in the current study, we investigated adult patients with CRS, with more attention on ECRS, for their CD40 and C40L levels, as well as ICOS and ICOSL levels. We characterized the clinical relevance of CD40-CD40L and ICOS-ICOSL, especially with eosinophils, in CRS, and we explored potential mechanisms that underlie their role in the pathogenesis of CRS.

## Material and methods

### Study subjects

We assessed 31 patients with CRS treated with functional endoscopic sinus surgery (FESS) from April 2021 to May 2021 in the otolaryngology department of The First Affiliated Hospital of Soochow University. The basic information and clinical characteristics of these patients are displayed in **Table 1**. The diagnosis of sinus disease was based on clinical symptoms and related examinations such as nasal endoscopy and CT, according to the guidelines of the European Position Paper on Rhinosinusitis and Nasal Polyps 2020 (EPOS2020) and the Chinese guidelines for diagnosis and treatment of CRS (2018). Participants whose age ranged from 18 to 70 were included. Our study excluded patients treated with oral, nasal, or systematic corticosteroids or antibiotics; patients treated antileukotrienes 4 weeks preceding the operation; patients suffering from upper respiratory tract infections 4 weeks preceding the operation; and patients developing immune disorders, pregnancy, malignancy such as nasopharyngeal carcinoma, and carcinoid such as inverting papilloma. At the same time, subjects who had CRS because of specific causes, cystic fibrosis, fungal sinusitis, vasculitis, or primary ciliary dyskinesia were excluded.

**Table 1:** Demographic and clinical profile of patients involved in the present study.

	ECRS	Non-eCRS
	10(9M/1F)	21(11M/10F)
**Age (years, mean ± std)**	39 ± 13	46 ± 15
**Patients with bilateral lesion, n (%)**	5 (50%)	10 (48%)
**Lund–Mackay score** **(mean ± std)**	13 ± 4	9 ± 4
**Eosinophils in PB** **(10^9^/L, mean ± std)**	0.37 ± 0.25	0.10 ± 0.08
Histological pattern		
** Edematous**	3 (30%)	1 (5%)
** Fibrotic**	0 (0%)	7 (33%)
** Hyperplasia**	0	8 (38%)
** Atypical**	0	0
** Edematous + fibrotic**	4 (40%)	3 (14%)
** Edematous + hyperplasia**	3 (30%)	2 (10%)
**CRSwNP, n (%)**	10 (100%)	17 (81%)
Comorbidity^*^		
** Atopy**	3/7 (43%)	5/17 (29%)
** Asthma**	1/7 (14%)	0/17 (0)
** Aspirin intolerance**	1/7 (14%)	1/17 (6%)

ECRS, eosinophilic chronic rhinosinusitis; non-eCRS, non eosinophilic chronic rhinosinusitis; M, male; F, female; std, standard deviation; CRSwNP, chronic rhinosinusitis with nasal polyp.

*Missing of clinical data.

Preoperative demographic information including sex, age, phone number, and drug allergies was obtained from each patient. Medical history including rhinorrhea, nasal blockage, hyposmia, facial pressure or pain, headache, duration, and prior nasal surgery was recorded carefully. Rhinology specialists classified CRS into CRSwNP and CRSsNP through nasal endoscopy and CT, into ECRS and Non-eCRS through the following hematoxylin–eosin (HE) staining. CT findings were graded according to the Lund–Mackay method. Blood samples were taken to perform complete blood cell counts. Recurrence of CRS was defined as the presence of NPs after nasal endoscopy. The study was approved by the ethics committee of The First Affiliated Hospital of Soochow University (No. 215).

### Histological analysis

Nasal tissues were obtained from ECRS (NPs) and non-eCRS (NPs or uncinate process), respectively. Tissues were immediately fixed in 10% formalin, embedded in paraffin, and cut into thin sections. Sections were stained with HE to differentiate CRS into various eosinophilic phenotypes. Representative HE staining pictures of non-eCRS and ECRS are shown in **Supplementary Figure 1A**. The numbers of eosinophils and total inflammatory cells beneath the epithelial surface per high-power field (HPF) (×400) were quantified by two independent researchers, and the percentage of eosinophils in total infiltrating inflammatory cells (eosinophils percentage) was calculated. Five fields were randomly selected, and then, the average percentage was analyzed. According to previous studies of ECRS in China, we defined ECRS as eosinophil percentage exceeding 10%, as proposed by Cao et al. (36).

At the same time, the histological patterns of each patient were evaluated according to histopathological characteristics referring to basement membrane thickening, goblet cell hyperplasia, subepithelial edema, submucous gland formation, eosinophils infiltration, fibrosis, and atypical cells by two independent researchers. Briefly, there were four main classifications: edematous: ECRS with a great number of eosinophils, goblet cell hyperplasia, and thickening of the basement membrane; CRS characterized by numerous seromucous glands and ductal structures; fibroinflammatory CRS: lack of stromal edema and goblet cell hyperplasia and frequently showed evident dilated vessels and a great number of fibrocytes; atypical CRS with distinct stromal cells that were bizarre and atypical. In addition, six patterns can be presented further: edematous, edematous + fibrotic, edematous + hyperplasia, fibrotic, hyperplasia, and atypical. We examined the HE data within four and six patterns to more thoroughly describe the histologic traits of patients with CRS. Representative HE stainings of the histologic pattern are shown in **Supplementary Figure 1B**.

### Immunohistochemistry analysis

For expression analysis of CD40, CD40L, ICOS, and ICOSL, formalin-fixed and paraffin-embedded nasal biopsies were cut into 4-μm-thick sections deparaffinized by serial treatment. Deparaffinized sections were subjected to antigen retrieval by heating the sections in sodium citrate buffer (pH 6.0). After blocking the endogenous peroxidase in 3% hydrogen peroxide and with 3% bovine serum albumin, the sections were incubated overnight at 4°C in the presence of rabbit-derived primary antibodies against CD40 (1:100; Affinity Biosciences, AF5336), CD40L (1:200; Abcam, Cambridge, MA, USA, ab65854), ICOS (1:500; Abcam, Cambridge, MA, USA, ab224644), and ICOSL (1:200; Abcam, Cambridge, MA, USA, ab233151). Thereafter, each section was incubated with HRP (horseradish peroxidase)–conjugated anti-rabbit secondary antibody (1:500) for 50 min. After washing, sections were incubated with 3,3′-diaminobenzidine tetrahydrochloride and immediately washed under tap water after color development. Then, sections were counterstained with hematoxylin and mounted with dibutyl phthalate xylene. The sections were blindly examined with no awareness of the clinical data with an Olympus CX40 Microscope (Olympus Optical Co., Hamburg, Germany). The average number of positive cells found in five randomly chosen HPFs (×200) was used to calculate the expression level.

### Immunofluorescence analysis

For further analysis of co-localization of CD40 and ICOSL with eosinophils, immunofluorescence was performed using TSA (Tyramide signal amplification) technique. Sections were deparaffinized, and antigen retrieval was performed in Tris–ethylenediaminetetraacetic acid buffer (pH 9.0). After blocking the endogenous peroxidase, sections were incubated overnight at 4°C in the presence of primary antibody against PRG2 (1:1,000; Abcam, Cambridge, MA, USA, ab236851), which is a major basic protein, the predominant constituent of the crystalline core of the eosinophil granule. Then, HRP-conjugated anti-rabbit secondary antibody (1:500) was incubated with sections for 50 min at room temperature. Sections were then incubated with 488 TSA at room temperature for 10 min. Next, antigen retrieval was performed before incubating with primary antibody against CD40 (1:250; Affinity Biosciences, AF5336) or ICOSL (1:200; Abcam, Cambridge, MA, USA, ab233151). After washing, sections were incubated with CY3-conjugated anti-rabbit secondary antibody (1:300). The DNA dye 4′,6-diamidino-2-phenylindole was used to visualize the nucleus. Results were captured under a fluorescence microscope. Agents not mentioned specifically obtained from Servicebio technology Co., Wuhan, China. For co-localization analysis between CD40 or ICOSL and eosinophils, the co-localization plugin of ImageJ software was used. Briefly, the RGB images of CY3 staining (CD40 or ICOSL) and 488 staining (PRG2) from a representative patient with ECRS were converted to gray-scaled images, and then, three regions of interest were selected, and the Manders’ co-localization coefficient M2, which defined as the proportion of co-localization component relative to the total CY3 fluorescent (CD40 or ICOSL) component in the same region, was calculated respectively. Finally, the mean M2 represented the relative percentage of eosinophils that express CD40 or ICOSL in all CD40- or ICOSL-positive cells (37).

### Assessment of blood eosinophils activation

Whole heparinized blood was obtained from 10 patients with ECRS. Blood was treated with red blood cell lysis buffer and then incubated for 24 h at 37°C with either recombinant human CD40L protein (rhCD40L, 5 µg/ml; R&D Systems, Minneapolis, MN, USA, 6420-CL-025) or recombinant human ICOS protein (rhICOS, 10 µg/ml; R&D Systems, Minneapolis, MN, USA, 169-CS-050). IgG (5 µg/ml; R&D Systems, Minneapolis, MN, USA, 1-001-A) was used as control. Cells were harvested for further analysis. Leukocytes were stained with an antibody cocktail of CD45-Allophycocyanin, APC (Life Technologies, CA, USA, 17-0459-42, HI30), CD16-Fluorescein isothiocyanate, FITC (BioLegend, San Diego, CA, USA, 360716, B73.1), and CD69-Phycoerythrin, PE (BioLegend, San Diego, CA, USA, 985202, FN50). Eosinophils were defined as CD45^+^CD16^−^, and CD69 was determined as its activation marker.

### Eosinophils isolation and culture

Peripheral blood eosinophils from healthy controls were purified by using an eosinophil isolation kit (Miltenyi Biotec, San Diego, CA, USA, 130-092-010). Eosinophil purity was assayed using flow cytometry and Wright–Giemsa staining (**Supplementary Figure 2A**). This procedure consistently resulted in a highly purified eosinophil population (95%–99%). These eosinophils (>99% viable by trypan blue exclusion) were cultured in RPMI 1640 medium supplemented with 10% fetal bovine serum (FBS), penicillin (100 U/ml), streptomycin (0.1 mg/ml), and granulocyte-macrophage colony-stimulating factor (GM-CSF, 50 ng/ml; Novoprotein, Suzhou, China, C003) at 37°C in a humidified atmosphere of 5% CO_2_. Then, eosinophils (2 × 10^5^ per well in 200 µl of RPMI) were stimulated in a 96-well plate for 24 or 48 h with or without the addition of the following agents: recombinant TNF-α (50 ng/ml; Novoprotein, Suzhou, China, C008), recombinant IL-5 (50 ng/ml; Novoprotein, Suzhou, China, CI59), 3 μM specific p38 mitogen-activated protein kinase (MAPK) inhibitor SB203580 (MedChemExpress, NJ, USA, HY-10256A), and 3 μM SB202474 (a negative analog of SB203580) (MedChemExpress, NJ, USA, HY-112367). At the end of this incubation, eosinophils were harvested and investigated further by using flow cytometry for the expression of CD40 and ICOSL.

### Flow cytometry analysis

Flow cytometry was used to detect CD40 and ICOSL expression on purified eosinophils at 0, 24, or 48 h. Every time when eosinophils were isolated, CD16 was used to access their purity. Briefly, harvested eosinophils were resuspended in phosphate buffer saline, PBS with 1% FBS. Cell suspension (100 μl) was incubated with the fluorescein-conjugated antibody at 4°C in the dark for 20 min. All the antibodies were purchased from BioLegend (San Diego, CA, USA), and the detailed information was as follows: CD16-FITC (360716, B73.1), PE anti-human CD40 (334308, 5C3), and APC anti-human ICOSL (309407, 2D3).

### Statistical analysis

All data were analyzed using GraphPad Prism 7 software (GraphPad, San Diego, CA, USA). Normality of variables was evaluated using Shapiro–Wilk test. Student’s unpaired t-test was performed for two-group comparisons of the data with normal distribution; otherwise, Mann–Whitney U-test was used. The analysis of variance (ANOVA) was performed for comparisons of multiple groups. In addition, the interaction between variables was assessed by Pearson’s/Spearman’s correlation test, which was appropriate for normally and abnormally distributed variables, respectively. P-values of less than 0.05 indicated statistical significance.

## Results

### CD40, ICOS, and ICOSL expressions are markedly increased in nasal tissues of patients with ECRS

**Figure 1:**
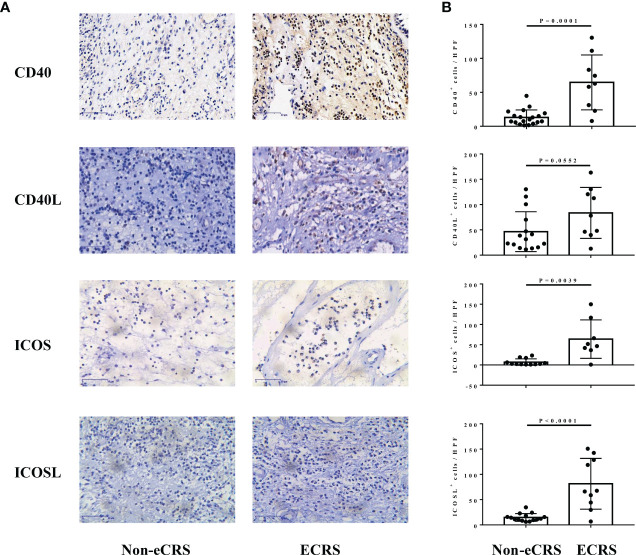
The expression of CD40, CD40L, ICOS, and ICOSL in nasal tissues of patients with ECRS and non-eCRS. **(A)** The representative immunohistochemistry stainings of CD40, CD40L, ICOS, and ICOSL. Original magnification, ×400. **(B)** The mean numbers of CD40^+^ (non-eCRS, n = 19; ECRS, n = 9), CD40L^+^ (non-eCRS, n = 15; ECRS, n = 9), ICOS^+^ (non-eCRS, n = 12; ECRS, n = 8), and ICOSL^+^ (non-eCRS, n = 15; ECRS, n = 10) cells in nasal tissues.

Representative staining of CD40, CD40L, ICOS, and ICOSL on sections from the nasal tissue involved in this study varied in density and intensity in patients with ECRS and non-eCRS (**Figure 1A**). The expression levels of CD40 (64.67 ± 13.48 *vs*. 13.12 ± 2.52, p = 0.0001), ICOS (63.85 ± 16.8 *vs*. 7.05 ± 2.31, p = 0.0039), and ICOSL (81.36 ± 15.88 *vs*. 14.72 ± 2.00, p < 0.0001) were significantly higher in the nasal tissues of patients with ECRS compared with that in patients with non-eCRS (**Figure 1B**). In addition, the number of CD40L-positive cells was also increased in ECRS nasal tissue compared with that in patients with non-eCRS, although there was no significant difference (**Figure 1B**).

### CD40-CD40L and ICOS-ICOSL expressions are correlated in nasal tissues of patients with CRS

**Figure 2:**
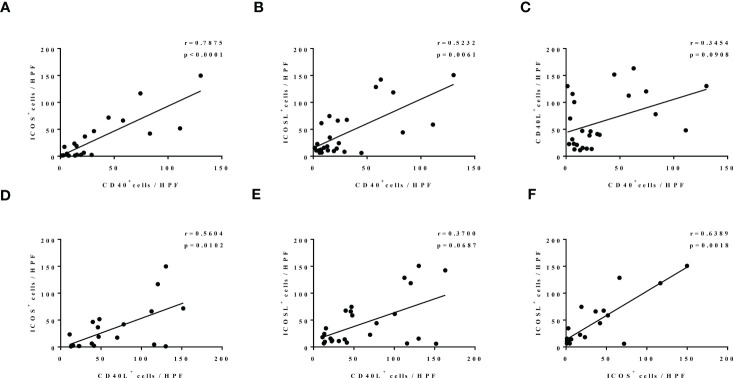
The correlation among the levels of CD40, CD40L, ICOS, and ICOSL in nasal tissues of patients with CRS. N = 21, 26, 25, 20, 25, and 21 in **(A–F)**.

Then, we investigated the correlation of CD40-CD40L and ICOS-ICOSL expression in the nasal tissues of patients with CRS. Our correlation analysis results show that there was a significantly positive correlation between ICOS and CD40 expression (r = 0.7875, p < 0.0001; **Figure 2A**), ICOSL and CD40 expression (r = 0.5232, p = 0.0061; **Figure 2B**), ICOS and CD40L expression (r = 0.5604, p = 0.0102; **Figure 2D**), as well as ICOS and ICOSL expression (r = 0.6389, p = 0.0018; **Figure 2F**). Similar correlation tendencies were observed between CD40L and CD40 expression (**Figure 2C**), as well as CD40L and ICOSL expression (**Figure 2E**), whereas there was no significant correlation shown.

### ICOSL expression is significantly higher in nasal tissues of patients with CRS with edematous pattern

**Figure 3:**
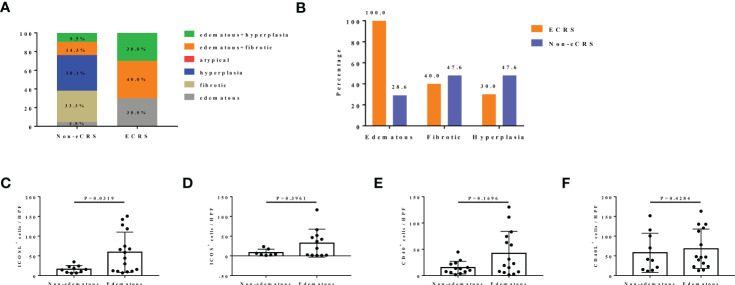
The expression of CD40-CD40L and ICOS-ICOSL in different histological patterns of patients with CRS. **(A)** The percentage of six different histological patterns in ECRS (n = 10) and non-eCRS (n = 21), respectively. **(B)** The percentage of three histological patterns (edematous: edematous, edematous + fibrotic, and edematous + hyperplasia; fibrotic: fibrotic and edematous + fibrotic; hyperplasia: hyperplasia and edematous + hyperplasia) in ECRS (n = 10) and non-eCRS (n = 21). **(C–F)** Expression levels of ICOSL (non-edematous, n = 7; edematous, n = 12), ICOS (non-edematous, n = 10; edematous, n = 16), CD40 (non-edematous, n = 13; edematous, n = 15) and CD40L (non-edematous, n = 10; edematous, n = 15) in nasal tissues of patients with edematous and non-edematous CRS.

All patients with CRS were also classified into different histopathological pattern. We found that the percentage of mere hyperplasia (38.1%) and fibrotic pattern (33.3%) were overwhelming in non-eCRS, whereas none of these two types were observed in ECRS (**Figure 3A**). In group ECRS, pattern edematous combined with fibrotic accounted for the largest proportion (40.0%), followed by edematous plus hyperplasia pattern (30.0%) and edematous pattern (30.0%), which were all characterized by edema (**Figure 3A**). When the six patterns were combined into three types (edematous: edematous, edematous + fibrotic, and edematous + hyperplasia; fibrotic: fibrotic and edematous + fibrotic; hyperplasia: hyperplasia and edematous + hyperplasia), the edematous pattern was seen in 28.6% of patients with non-eCRS and 100.0% in patients of ECRS. Whereas, the proportions of fibrotic and hyperplasia patterns were both slightly lower in ECRS than non-eCRS, respectively (40.0% *vs*. 47.6%, 30.0 *vs*. 47.6%; **Figure 3B**).

According to the great difference of the proportion in edematous subtype and merely little variation of that in hyperplasia and fibrotic subtypes between ECRS and non-eCRS, we thus only examined CD40-CD40L and ICOS-ICOSL expression in the histopathological subtype of edema. Results showed that the expression levels of ICOSL (59.21 ± 12.76 *vs*. 16.16 ± 2.89, P = 0.0319; **Figure 3C**) were significantly increased in the nasal tissues of patients with CRS with edematous pattern compared with non-edematous pattern. The number of ICOS- or CD40-positive cells was also higher in edematous pattern compared with that in non-edematous pattern, but no significant difference was observed (**Figures 3D, E**). In contrast, there was no obvious difference of CD40L expression in nasal tissues between edematous and non-edematous patterns (**Figure 3F**).

### CD40-CD40L and ICOS-ICOSL expression are strongly correlated in nasal tissues of patients with edematous pattern CRS

**Figure 4:**
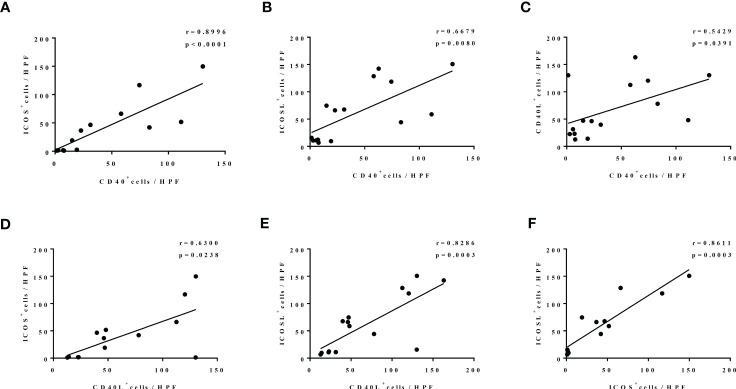
The correlation among the levels of CD40, CD40L, ICOS, and ICOSL nasal polyp of patients with edematous CRS. N = 13, 15, 15, 13, 15, and 13 in **(A–F)**.

In addition, we further found a strong positive correlation between the expression of CD40-CD40L and ICOS-ICOSL in nasal tissues of patients with CRS with edematous pattern. To be specific, there was a significantly positive correlation between ICOS and CD40 expression (r = 0.8966, p < 0.0001; **Figure 4A**), ICOSL and CD40 expression (r = 0.6679, p = 0.0080; **Figure 4B**), CD40L and CD40 expression (r = 0.5429, p = 0.0391; **Figure 4C**), ICOS and CD40L expression (r = 0.6300, p = 0.0238; **Figure 4D**), ICOSL and CD40L expression (r = 0.8286, p = 0.0003; **Figure 4E**), as well as ICOSL and ICOS expression (r = 0.8611, p = 0.0003; **Figure 4F**).

### High levels of CD40-CD40L and ICOS-ICOSL expression in nasal tissues are linked to high eosinophil levels and disease activity in patients with CRS

**Figure 5:**
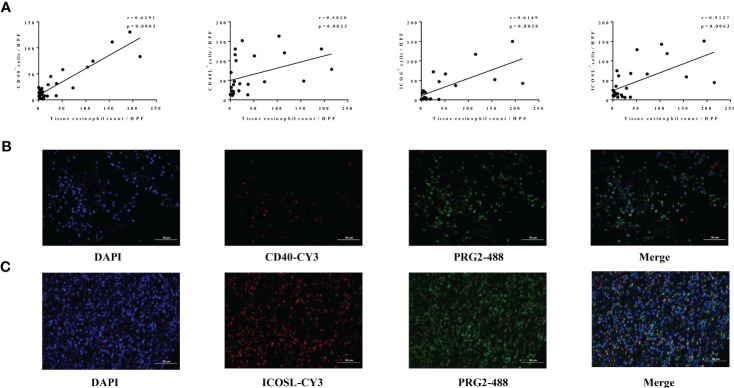
Association between levels of CD40-CD40L, ICOS-ICOSL, and eosinophil in nasal tissues of CRS. **(A)** The correlation analysis between the number of CD40^+^ (n = 28), CD40L^+^ (n = 25), ICOS^+^ (n = 21), ICOSL^+^ (n = 27) cells, and tissue eosinophils levels in CRS. **(B, C)** The co-localization of eosinophils (PRG2, 488) and CD40 (CY3) as well as ICOSL (CY3) assessed by immunofluorescence in patients with ECRS. Original magnification, ×400.

Our further findings showed that the expression levels of CD40 (r = 0.6291, p = 0.0003), CD40L (r = 0.5820, p = 0.0023), ICOS (r = 0.6149, p = 0.0030), and ICOSL (r = 0.5127, p = 0.0063) in nasal tissues of patients with CRS were all significantly correlated with tissue eosinophil count (**Figure 5A**). Consistently, our immunofluorescence co-staining results showed that the mean M2 referring to CD40 or ICOSL were 0.871 ± 0.110 and 0.871 ± 0.033, respectively, which demonstrated that a great number of CD40-positive cells and ICOSL-positive cells were eosinophils in ECRS nasal tissues (**Figures 5B, C**).

**Figure 6:**
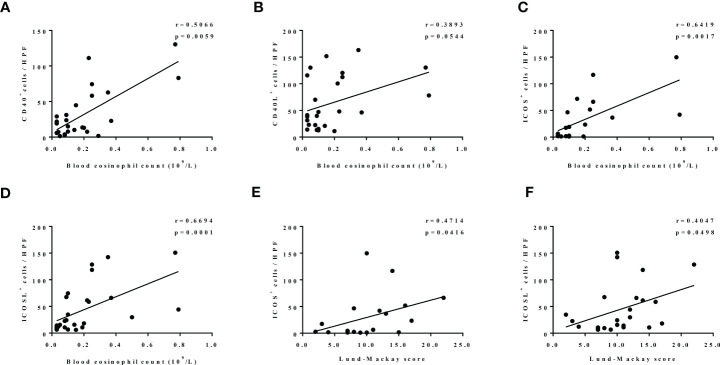
**(A–D)** The correlation between expression levels of nasal tissues CD40 (n = 28), CD40L (n = 25), ICOS (n = 21), ICOSL (n = 27), and blood eosinophil count. **(E, F)** The correlation between expression levels of ICOS (n = 19) and ICOSL (n = 24) in nasal tissues and Lund–Mackay score.

Furthermore, both increased CD40 and CD40L expression in our patients with CRS were linked to higher blood eosinophil count (r  = 0.5066, p = 0.0059; **Figure 6A**; r  = 3893, p = 0.0544; **Figure 6B**). Similarly, ICOS- and ICOSL-positive cell numbers were strongly positively correlated with blood eosinophil count (r = 0.6419, p = 0.0017; **Figure 6C**; r = 0.6694, p = 0.0001; **Figure 6D**). Moreover, ICOS and ICOSL expression levels correlated with disease activity assessed by Lund–Mackay score (r = 0.4714, p = 0.0416; **Figure 6E**; r = 0.4047, p = 0.0498; **Figure 6F**).

### CD40-CD40L and ICOS-ICOSL interactions enhance the activation of eosinophils from patients with ECRS

**Figure 7:**
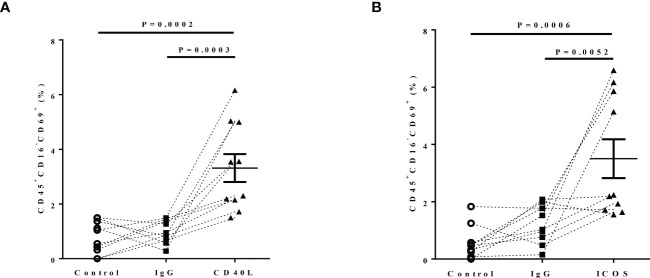
The effect of CD40-CD40L and ICOS-ICOSL pathways on eosinophil activation. Peripheral blood of patients with ECRS was stimulated with medium alone (Control, circle, n = 5), IgG (5 µg/ml, rectangle, n = 7), **(A)** rhCD40L (5 µg/ml, triangle, n = 10) or **(B)** rhICOS (10 µg/ml, triangle) for 24 h. Then, CD69 expression on eosinophils was detected by flow cytometry. Activated eosinophils were defined as CD45^+^CD16^−^CD69^+^ cells. The percentage of activated eosinophils after different stimulation.

Because upregulation of CD40 and ICOSL expression in nasal tissues of patients with ECRS, which mainly located on eosinophils, we investigated whether CD40-CD40L and ICOS-ICOSL interactions involved in eosinophils dysfunction. Considering that ECRS is characterized by both circulating and histologically high proportions of eosinophils. To determine this, peripheral blood samples from 10 patients with ECRS were stimulated by rhCD40L (5 µg/ml), rhICOS (10 µg/ml), or control IgG (5 µg/ml), respectively. Cells were harvested 24 h after stimulation for flow cytometry. CD45^+^C16^−^ cells were defined as eosinophils, and CD69 was an activation marker of eosinophils. There was notable upregulation of CD69 expression on eosinophils in response to CD40L (**Figure 7A**) and ICOS (**Figure 7B**) protein stimulation compared with that to control or IgG group. These data indicated that the upregulation of CD40 and ICOSL on eosinophils mediated their activation in patients with ECRS.

### TNF-α plus IL-5 enhanced CD40 expression on eosinophils depending on the activation of the p38 MAPK pathway

**Figure 8:**
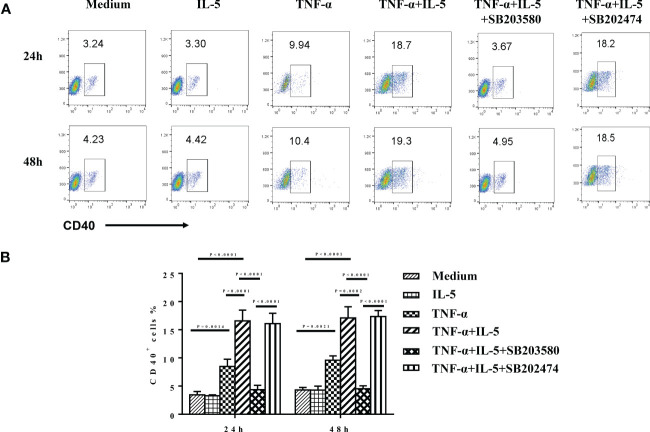
The effect of TNF-α and IL-5 on CD40 expression of purified eosinophils. GM-CSF (50 ng/ml) was added as a basic condition (medium) to keep eosinophils culture for all groups. Freshly isolated eosinophils from healthy controls were cultured with medium alone, rhTNF-α (50 ng/ml), rhIL-5 (50 ng/ml), or rhTNF-α plus rhIL-5 in the absence or presence of specific p38 inhibitor SB203580 (3 μM) and negative analog SB202474 (3 μM) for 24 and 48 h. In addition, SB203580 and SB202474 were preincubated with eosinophils for 1 h before cytokines stimulation. CD40 expression was subsequently detected by flow cytometry. **(A)** The expression level of CD40 in the different conditions detected by flow cytometry. **(B)** The ANOVA comparisons among different conditions.

Finally, we investigated the possible inflammatory mediators involved in enhanced CD40 and ICOSL expression on eosinophils in patients with CRS. As CRS is characterized by the increased local tissue levels of TNF-α and IL-5, especially in ECRS (38–40). Previous studies have reported that TNF-α induces the expression of CD40 on epithelial and endothelial cells as well as the expression of ICOSL expression on fibroblasts, endothelial cells, B cells, and monocytes (41–45). IL-5 is the most potent activator of eosinophils (46–48). Thus, we investigated the effect of TNF-α and IL-5 on CD40 and ICOSL expression on human eosinophils. At the baseline, purified eosinophils from healthy human peripheral blood (purity > 95%) have no CD40 or ICOSL expression (**Supplementary Figure 2B**). As shown in **Figures 8A, B**, the expression of CD40 was markedly upregulated on eosinophils after rhTNF-α (50 ng/ml) stimulation (P = 0.0014) for 24 h but not rhIL-5 (50 ng/ml). Furthermore, TNF-α plus IL-5 further markedly enhanced CD40 expression on eosinophils compared with TNF-α incubation (p < 0.0001). However, no time-dependent effects of TNF-α or TNF-α plus IL-5 on CD40 expression of eosinophils were observed. Whereas, TNF-α alone or TNF-α combined with IL-5 has no significant effect on ICOSL expression on eosinophils (**Supplementary Figures 2C, D**).

Activation of p38 MAPK has been shown to partly mediated TNF-α–induced anti-apoptotic signals in human eosinophils (49). Finally, we sought to determine whether p38 MAPK pathway mediates the up-expression of CD40 here. Purified eosinophils were treated with 3 μM SB203580 and SB202474 before TNF-α plus IL-5 stimulation. (Because there was no difference in the pre-experiment among the treatment with 3, 10, or 30 μM SB203580, we decided to use 3 μM as the final concentration. Data are not shown.) We found that SB203580 highly suppressed the TNF-α + IL-5–induced CD40 expression on eosinophils (p < 0.0001; **Figures 8A, B**).

## Discussion

This is the first study to show that both CD40-CD40L and ICOS-ICOSL are upregulated in the NPs of patients with ECRS. Our results demonstrate that increased expression of CD40-CD40L and ICOS-ICOSL in CRS nasal tissues is linked to high eosinophils infiltration and disease severity. Then, we found CD40-CD40L and ICOS-ICOSL pathways do take effect on the activation of eosinophils from patients with ECRS. In addition, we illustrated that TNF-α induces CD40 expression on eosinophils *via* the activation of the p38 MAPK signaling pathway, and IL-5 further augments TNF-α–stimulated CD40 expression on eosinophils. Our findings indicate that CD40-CD40L and ICOS-ICOSL are potential clinical biomarkers of disease activity in patients with CRS, particularly in the population with high-level eosinophils.

For the first time, our findings show that the levels of CD40-CD40L and ICOS-ICOSL are markedly increased in the nasal tissue of patients with ECRS compared with that in patients with non-eCRS. Our subsequent correlation analyses showed that high nasal tissue CD40-CD40L and ICOS-ICOSL levels were strongly correlated in CRS. Moreover, on the basis of the classification of histopathologic phenotypes, we observed similar upregulation of CD40 and ICOS-ICOSL in nasal tissues of edematous CRS. Consistently, a strong correlation with CD40-CD40L and ICOS-ICSOL levels was observed in edematous CRS nasal tissues. It has been reported that edematous CRS was commonly observed in eosinophilic inflammation (1, 50). Edema pattern is more likely to develop in such an immune microenvironment where CD40-CD40L and ICOS-ICOSL as well as specialized cells like eosinophils, Th2, and ILC2 may play a more significant role than non-eosinophilic inflammation. The higher level of co-stimulators and stronger correlation among them can be the predictor of this pattern. However, our findings that groupings based on ECRS/non-eCRS and groupings based on histopathologic phenotypes do not completely overlap. We think that this is mainly related to the uneven distribution and the small number of patient cases in different pathological subtypes, so statistical analysis cannot be conducted. In addition, age may further contribute to this inconsistency. Although we selected adult patients to minish age-related interference, there may be altered immune response and different distributions of inflammatory mediators in further classifications of adults (51–54). Given that the ICOS-ICOSL signal can strengthen CD40-CD40L interaction, thus providing a co-stimulatory signal for B-cell activation (34, 35), as well as the allergic characteristics of CRS. Importantly, our findings indicate that high CD40-CD40L and ICOS-ICOSL expressions in nasal tissues are potential immunoregulatory factors for the development of CRS, especially in patients with high eosinophil levels.

Then, our subsequent correlation analyses showed that high CD40-CD40L and ICOS-ICOSL expression was linked to high eosinophils infiltration in the nasal tissue of patients with CRS. We further observed that both augmented CD40 and ICOSL expressions were primarily on eosinophils in the local tissue of ECRS.

So far, several studies have shown that not only CD40 but also CD40L is expressed on the surface of human eosinophils (24, 55, 56). In addition, close to our results, Ohkawara et al. also found that CD40 was mainly expressed on the surface of eosinophils in the NP tissues of allergic subjects. They only compared the expression of CD40 in NP tissues (24). In this study, we further found the different CD40-CD40L expression in non-ECRS and ECRS nasal tissues and also their correlation with clinical feature of CRS. We assume that the high nasal tissue eosinophil proportion of CRS mainly contributed to the high CD40 expression levels. As CD40L is predominantly expressed on activated CD4^+^ T cells, it has been shown that there is a large number of T cells infiltrating in nasal tissue of CRS (36, 57–59). Thus, we suspect that there is a “T-eosinophils–centered function” of CD40-CD40L in the nasal tissue of CRS with high-level eosinophils, which is worthy of further study.

As for the expression of ICOS-ICOSL in nasal tissues, we reported it for the first time. Hutloff et al. reported that there is no ICOS expression on granulocytes using F44 (specific monoclonal antibody to ICOS) (60). In addition, no research has studied the expression of ICOSL on eosinophils so far. Our co-localization staining showed first ICOSL on eosinophils. Considering that ICOS is mainly expressed on activated CD4^+^T cells, especially activated Th2 cells (61, 62). ECRS found worldwide is characterized by a type 2 immune response involving Th2 cells, type 2 innate lymphoid cells, eosinophils, mast cells, and M2 macrophages (59, 63–67). Thus, we speculate that activated CD4^+^ cells, especially Th2 cells, can exert influence on eosinophils mono-directionally, through ICOS-ICOSL ligation signal pathway in ECRS. Further studies are still needed.

Then, our clinical correlation analysis shown that blood eosinophils count was significantly higher in ECRS subset compared with that in non-eCRS subset (**Supplementary Figure 3A**), which is consistent with previous studies (68–70) However, there was no significant difference of Lund-Mackay score, blood neutrophil count and blood basophil count between ECRS and Non-eCRS (**Table 2**). At the same time, the Lund-Mackay score and tissue eosinophil count showed no statistical correlation (**Table 3**). As shown in **Supplementary Figures 3B, C**, we further observed that blood eosinophil count was positively correlated with disease activity assessed by Lund–Mackay score as well as nasal tissue eosinophils count in our patients with CRS. Developing from progenitors in bone marrow, eosinophils can be recruited to diseased nasal tissue from peripheral circulation by chemokines and cytokines, resulting in a specific correlation between them. Then, positive correlations between blood eosinophil count and tissue CD40-positive cell numbers as well as CD40L-positive cell numbers were found, and the same findings were with ICOS-ICOSL–positive cell numbers. Importantly, we noticed that high ICOS-ICOSL expression levels were positively correlated with Lund–Mackay score of patients with patients with CRS. Although, there is still non-significant correlation between the expression level of CD40, CD40L, ICOS, ICOSL and clinical indexes such as Lund-Mackay, blood neutrophil count, blood basophil count (**Table 3**). Recent studies have reported the pathological effect of ICOS-ICOSL signals widely participate in inflammatory responses, particularly ICOS^+^ T cells, including Th1, Th2, and Th17 as well as T follicular helper (Tfh), T follicular regulatory cells (Tfr), and regulatory T cells (Treg), with the increased generation, proliferation, and survival abilities (71–75). Thus, the ICOS-ICOSL pathway may associate with the local immune microenvironment and then contribute to the development of CRS, especially ECRS. Interestingly, ICOSL-positive cells also had positive correlation with blood basophils (**Supplementary Figure 3D**). Therefore, our above data indicate that CD40-CD40L and ICOS-ICOSL signals may involve in the pathogenies of CRS by modulating the function of eosinophils.

**Table 2:** Unpaired T-test between patients with non-eCRS and ECRS.

	Non-eCRS (n = 21)	ECRS (n = 10)	P-value
**Lund–Mackay score**	9.18 ± 4.32	12.60 ± 4.09	0.053
**Blood neutrophil count (10^9^/L)**	4.20 ± 1.30	4.45 ± 1.62	0.650
**Blood basophil count (10^9^/L)**	0.03 ± 0.02	0.04 ± 0.02	0.279

Values were expressed as mean ± standard deviation.

**Table 3:** Correlation analysis of CD40-CD40 and ICOS-ICOSL expression and clinical parameters in patients with CRS.

Parameter 1	Parameter 2	n	R-value	P-value
Lund–Mackay score	Tissue eosinophil count/HPF	27	0.3042	0.1229
CD40^+^ cells/HPF	Lund–Mackay score	24	0.2300	0.2795
CD40^+^ cells/HPF	Blood neutrophil count (10^9^/L)	28	−0.2830	0.1445
CD40^+^ cells/HPF	Blood basophil count (10^9^/L)	28	0.0581	0.7689
CD40L^+^ cells/HPF	Lund–Mackay score	22	0.1168	0.6048
CD40L^+^ cells/HPF	Blood neutrophil count (10^9^/L)	25	0.1431	0.4951
CD40L^+^ cells/HPF	Blood basophil count (10^9^/L)	25	0.0376	0.8584
ICOS^+^ cells/HPF	Blood neutrophil count (10^9^/L)	21	−0.2859	0.2090
ICOS^+^ cells/HPF	Blood basophil count (10^9^/L)	21	0.2881	0.2054
ICOSL^+^ cells/HPF	Blood neutrophil count (10^9^/L)	27	−0.0907	0.6529

Next, we confirmed whether CD40-CD40L and ICOS-ICOSL axes function on eosinophils by using CD40L and ICOSL protein in ECRS. We found that CD40L protein stimulation upregulated the expression of CD69, which is an important marker of activation for eosinophils. In addition, CD69 levels were also increased in response to ICOSL protein stimulation. These results show that both CD40-CD40L and ICOS-ICOSL signals activate eosinophils and then contribute to the development of ECRS. Recent evidence suggests that activated eosinophils have an axial role in symptomology of CRS, especially ECRS. Studies have shown the association between activated eosinophil count and the development of ECRS. Moreover, some reports demonstrated a significant drop of blood eosinophils from before to after FESS (70, 76–78). In the advantage of great local cytokines and chemokines production, eosinophils are characterized by increased production, enhanced activation, and prolonged survival. These factors promote the eosinophils accumulation, ultimately contributing to the increased destroy of epithelial barrier and hyper-activity in nasal mucosa (79–81). In addition to activation of eosinophils, the survival and recruitment to inflammatory site are both crucial for the development of nasal inflammatory. In fact, studies have demonstrated that activation of CD40-CD40L pathway increased eosinophil survival *via* induction of GM-CSF release and cellular inhibitor of apoptosis protein 2 (55). The trafficking of eosinophils from blood into inflammatory nasal cavity involves repeated adhesion and detachment among endothelial cells, epithelial cells, intercellular matrix, and eosinophils, in which adhesion molecules including intercellular adhesion molecule 1 (ICAM-1) and vascular cell–adhesion molecule (VCAM-1) are regulated constantly (82–84). Several groups found that a trimeric form of recombinant murine CD40 ligand induced the expression of leukocyte adhesion molecules, such as E-selectin, VCAM-1, and ICAM-1 on human vascular endothelial cells (22, 41, 85). At the same time, chemokines including eotaxin, RANTES, monocyte chemoattractant protein (MCP), and macrophage inflammatory protein (MIP) also regulate eosinophils infiltration (86, 87). Some research studies have demonstrated that CD40 ligation can induce expression of IL-8, MCP-1, RANTES in fibroblasts, epithelial cells, and endothelial cells (88–90). In addition, at present, the chemotaxis of ICOS signal only limited in T cells by a way to regulate the expression of chemokine receptor. Research studies showed that ICOS ligand enhanced the homing of Tfh to the follicular region through the induction of C-X-C motif chemokine receptor 5 (CXCR5) as well as the chemotaxis of Treg to pancreas islet through CXCR3 in in early phase of diabetes (91, 92). However, there is no any study about CD40-CD40L or ICOS-ICOSL mediated eosinophils recruitment. All these results show the possible different roles of co-stimulators in eosinophils trafficking and the great potent to study further.

TNF-α and IL-5 are closely related to CRS. Previously, many researchers have reported the high levels of TNF-α and IL-5 in patients with CRS and positive correlation with disease activity (38, 39). In addition, TNF-α and IL-5 are critical for the function of eosinophils including antigen presentation, cytokine or chemokine production, and secretion of granule mediators (47, 93, 94). Furthermore, clinical studies of anti–IL-5 antibody (Ab) and anti–IL-5 receptor (IL-5R) Ab have been performed for severe CRSwNP. Several placebo-controlled double-blind study of anti–IL-5 (mepolizumab) and anti–IL-5RA (benralizumab) demonstrated to decrease NPs and to improve CT findings in patients with large NPs, especially in ECRS (95–97). Then, we observed that TNF-α stimulation significantly upregulated CD40 expression on eosinophils, which was further markedly enhanced by combined incubation with IL-5. However, TNF-α, IL-5, or TNF-α plus IL-5 stimulation feebly affected ICOSL expression on eosinophils, no significant difference was observed compared with that in control groups. These results indicated that TNF-α and IL-5 mainly affected the expression of CD40 on eosinophil. As for the expression of ICOSL-derived eosinophils, the specific mechanism needs to be further explored in the future. For example, is there a synergistic effect of cytokines? Or other potential, unknown mediators? In fact, there are other Th2 cytokines that play a pivotal role in the eosinophilic inflammatory including IL-4 and IL-13. In part, because of shared receptor affinity for IL-4Rα, IL-4 and IL-13 have overlapping roles (98). Some studies have demonstrated that IL-4 and IL-13 can enhance eosinophils survival and activation assessed by CD69 expression (99, 100). Furthermore, eosinophils infiltration in local inflammatory site can be promoted by IL-4 and IL-13, through eosinophil chemotaxis induction and increased adhesiveness between endothelial cells and eosinophils (99, 101–103). In clinical trials, targeting on IL-4 and IL-13 has been a promising potential biologic therapy for CRS. A randomized control trial that examined the effects of dupilumab, a monoclonal antibody to the α subunit of the IL-4 receptor (IL-4Rα) that inhibits signaling of IL-4 and IL-13, in patients with CRSwNP versus placebo have demonstrated significant improvements in polyp size, disease-specific SNOT-22 (22-item Sinonasal Outcome Test) score, and objective olfactory function (104). Furthermore, there are other clinical trials of immunotherapy targeting eosinophils including CSL311(a novel human monoclonal antibody that may interact with GM-CSF/IL-3/IL-5 at the same time, specifically targeting eosinophil survival) and anti–Siglec-8 antibody [sialic acid–binding immunoglobulin-like lectin 8 is a surface receptor predominantly expressed on human eosinophils where its ligation induces reactive oxygen species (ROS) formation and cell death] (105–107). Thus, we think that whether these cytokines like IL-4, IL-13, GM-CSF/IL-3, and molecule such as siglect-8 regulate the function of eosinophils through CD40-CD40L or ICOS-ICOSL pathway can be further studied.

Since previously, it has been described that p38 MAPK is activated in eosinophils by TNF-α (49, 93). Thus, in discerning the individual contributions of specific signaling pathways, we observed the effect of the inhibitor that targeted the p38 MAPK pathway. The present study shows that the specific p38 MAPK inhibitor SB203580 could largely inhibit TNF-α plus IL-5–induced CD40 expression on eosinophils. These data indicated the important role of the activation of p38 MAPK in the mechanism of TNF-α plus IL-5–induced CD40 expression on eosinophils. Therefore, modulation of TNF-α/IL-5/CD40/p38 MAPK pathways might be useful for the treatment of CRS. Moreover, we found that SB203580 did not fully inhibit the CD40 expression on eosinophils. These findings indicate that pathways other than p38 MAPK are also involved in TNF-α– and IL-5–induced inhibition of CD40 expression on eosinophils. Because p38 MAPK is required for NF-kB–dependent gene expression and CD40 gene expression could partly mediated by nuclear factor kappa-B, NF-kB, it is reasonable that the inhibition of p38 MAPK can downregulate the expression of CD40 (108–111). Therefore, it may be possible that the inhibition of p38 MAPK by SB203580 can block TNF-α– and IL-5–induced eosinophil-derived CD40 by indirect inhibiting NF-kB activity and subsequently suppress the eosinophil activation. Further investigation is required to explore other signaling pathways involved in TNF-α– and IL-5–mediated modulation of CD40 expression on eosinophil.

Critical roles for CD40-CD40L signaling include promoting antigen presentation and B- and T-cell priming in a range of inflammatory responses. As such, abnormalities in the CD40-CD40L co-stimulation pathway are frequently observed in a variety of human diseases. Many studies have found increased serum or plasma levels of soluble CD40L (sCD40L) in patients suffering from systemic lupus erythematosus (SLE), Sjögren’s syndrome (SS), inflammatory bowel disease (IBD), and cardiovascular disease (112–117). Being a biomarker, plasma levels of sCD40L correlate with anti–double-stranded DNA (dsDNA) titers and disease severity in patients with lupus (112, 118). In addition, increased sCD40L indicates an increased risk of cardiovascular events and susceptibility for vascular damage in patients with cardiovascular disease (113, 117). At present, there are exciting data on the anti-CD40L treatment efficacy referring to transplantation, SLE, and immune thrombocytopenic purpura (ITP) (119, 120). For example, in a phase II trial in lupus nephritis, efficacy of ruplizumab was supported by the reduced symptoms, reductions in proteinuria, anti-dsDNA antibodies, and hematuria (121, 122). At the same time, as the obstruction on future clinical experience, the complication of thromboses should be paid more attention.

As the co-stimulator regulating the proliferation, differentiation, and effective function of T cells, especially Treg and Tfh, the upregulation or downregulation of ICOS is closely correlated with the development of autoimmune diseases. In the animal model of no obesity diabetes, ICOS promoted the development of hyperglycemia through the increased production of IFN-γ by Th1 while sustaining the function and homeostasis of Treg cells (91, 92). Studies have showed that there is higher ICOS expression level in patients with SLE. In the experimental animal in which the gene encoding ICOSL was specifically deleted, the symptoms including proteinuria and interstitial nephritis, the infiltrated effective T cells, as well as the level of autoantibody decreased compared to the control (123, 124). Furthermore, through abnormal regulation of Tfh cell function, ICOS is involved in the development and deterioration in some diseases like rheumatoid arthritis (RA), myasthenia gravis, and multiple sclerosis (MS) (125–127). Thus, interfering with the ICOS signaling pathways may be a potential treatment for autoimmune diseases. Therefore, targeting CD40-CD40L and ICOS-ICOSL has a significant therapeutic potential for treating chronic inflammation, such as CRS.

The limitations of our study are its retrospective, cross-sectional design, the univariate and descriptive nature of the analyses performed, the lack of a large cohort of patients with CRS, and the not yet identified relevant mechanisms underlying including the regulation of the survival and recruitment of eosinophils through CD40-CD40L and ICOS-ICOSL pathway.

In summary, we observed that the high levels of CD40-CD40L and ICOS-ICOSL in local nasal tissues are closely associated with high eosinophils infiltration and high disease activity in CRS. We demonstrated a previously unrecognized role for CD40-CD40L and ICOS-ICOSL pathways, most remarkably in eosinophil activation of ECRS. Our data have shown that TNF-α and IL-5 mediate CD40 upregulation in human eosinophils in part *via* activation of p38 MAPK. In view of the above findings, we conclude that blocking of the activation of eosinophils by targeting CD40-CD40L and ICOS-ICOSL pathways, especially manipulation of TNF-α/p38 MAPK pathways targeting eosinophils activation might be useful for the treatment of CRS with high-level eosinophils.

## Data availability statement

The original contributions presented in the study are included in the article/**Supplementary Material**. Further inquiries can be directed to the corresponding authors.

## Ethics statement

The studies involving human participants were reviewed and approved by the first affiliated hospital of Soochow University. The patients/participants provided their written informed consent to participate in this study.

## Author contributions

AZ has analyzed the samples and was involved in statistical analysis, manuscript preparation, and proofreading of the manuscript. CS and YZ have coordinated the histological pathology part of the study. YF, JW, ZL and HX have coordinated the study and collected patient data. JL was involved in statistical analysis and proofreading of the manuscript. QJ was the overall study coordinator and was involved in manuscript preparation and proofreading of the manuscript. All authors contributed to the article and approved the submitted version.

## Supplementary material

Supplementary Figure 1 Representative eosinophilic subtypes and histological patterns in nasal tissues of CRS patients. **(A)** Eosinophils infiltration in nasal tissue of ECRS and Non-eCRS. Eosinophils were in the bottom of right panel. **(B)** Different histological change in nasal tissue of 4 patterns: edematous CRS with a great number of eosinophils (thick arrow), goblet cell hyperplasia (triangle), thickening of the basement membrane (arrow), and the loose stroma contains pseudocystic spaces filled with fluid (star); CRS with hyperplasia of seromucinous glands (arrow); Fibroinflammatory CRS with evident dilated vessels(star) and a great number of fibrocytes (arrow); Atypical CRS with bizarre cells in stroma. The nuclei of these “atypical” cells often tend to be hyperchromatic (arrow). Original magnification x200.

Supplementary Figure 2 The effect of TNF-α and IL-5 on ICOSL expression of purified eosinophils. **(A)** The purity of isolated eosinophils verified by Flow cytometry (left panel) and Wright–Giemsa staining (right panel, x 1,000). **(B)** The expression level of CD40 and ICOSL on purified eosinophils at 0h accessed by flow cytometry. **(C)** The effect of TNF-α and IL-5 on ICOSL expression of eosinophils isolated from healthy donators. GM-CSF (50 ng/mL) stimulation used as basic condition (medium) for all groups. Freshly isolated eosinophils were cultured with medium alone, or rhTNF-α (50 ng/mL), or rhIL-5 (50 ng/mL) or rhTNF-α plus rhIL-5. ICOSL expression was then detected by Flow cytometry.

Supplementary Figure 3
**(A)** The blood eosinophil count in ECRS (n=10) and Non-eCRS (n=21). **(B)** The correlation between blood eosinophil count and Lund-Mackay score (n=27). **(C)** The correlation between tissue eosinophil count and blood eosinophil count (n=31). **(D)** The correlation between blood basophil count and the levels of ICOSL in nasal tissue of CRS patients (n=27).
