# Supplementary material for: Involvement of CD40-CD40L and ICOS-ICOSL in the development of chronic rhinosinusitis by targeting eosinophils
Source: Front Immunol. 2023 Jun 1;14:1171308. doi: 10.3389/fimmu.2023.1171308 (PMC10267736; doi:10.3389/fimmu.2023.1171308)
Supplement: Supplementary Figure 1 — Representative eosinophilic subtypes and histological patterns in nasal tissues of CRS patients. (A) Eosinophils infiltration in nasal tissue of ECRS and Non-eCRS. Eosinophils were in the bottom of right panel. (B) Different histological change in nasal tissue of 4 patterns: edematous CRS with a great number of eosinophils (thick arrow), goblet cell hyperplasia (triangle), thickening of the basement membrane (arrow), and the loose stroma contains pseudocystic spaces filled with fluid (star); CRS with hyperplasia of seromucinous glands (arrow); Fibroinflammatory CRS with evident dilated vessels(star) and a great number of fibrocytes (arrow); Atypical CRS with bizarre cells in stroma. The nuclei of these “atypical” cells often tend to be hyperchromatic (arrow). Original magnification x200. [file DataSheet_1.pdf]

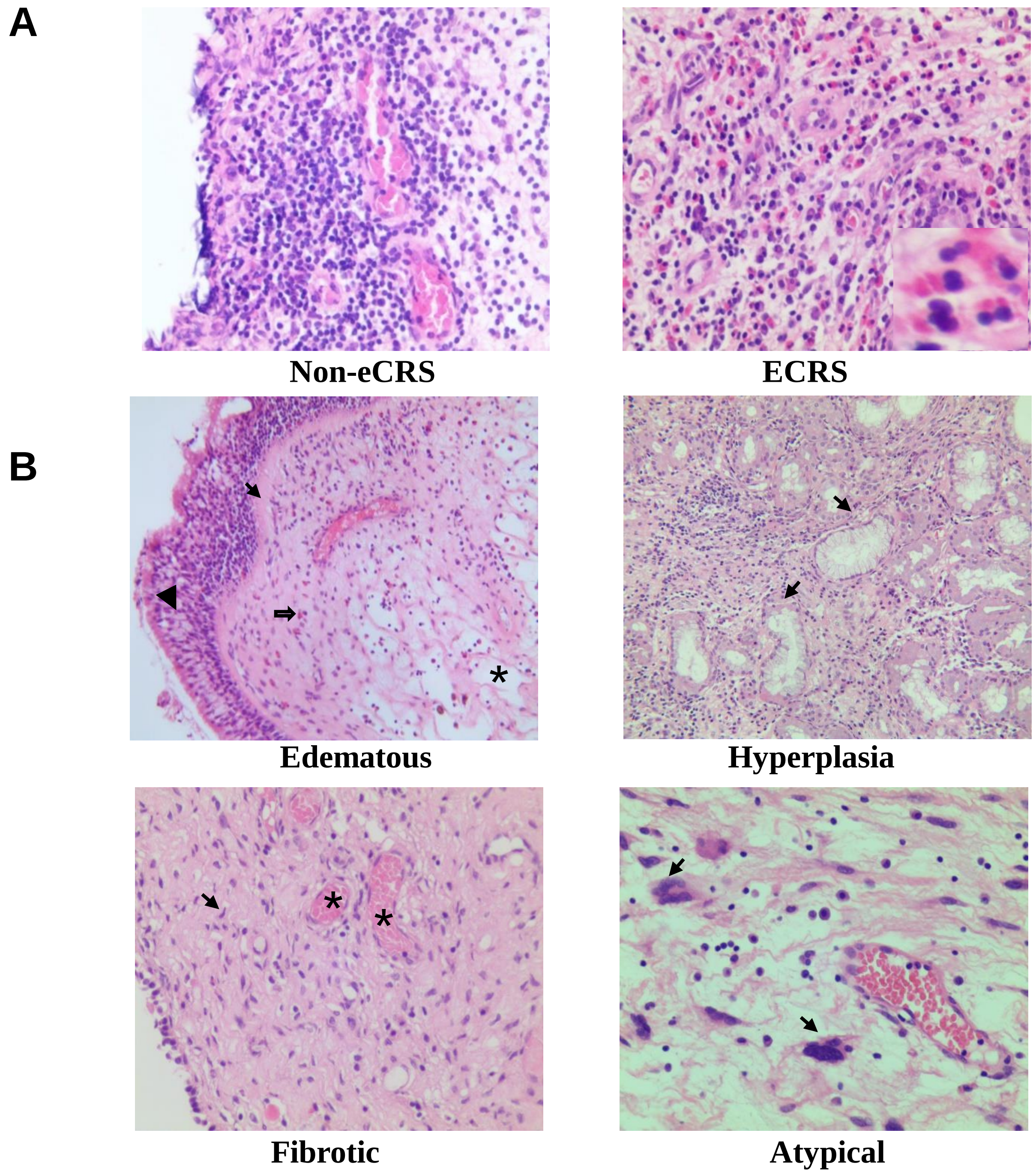

Supplementary figure 1

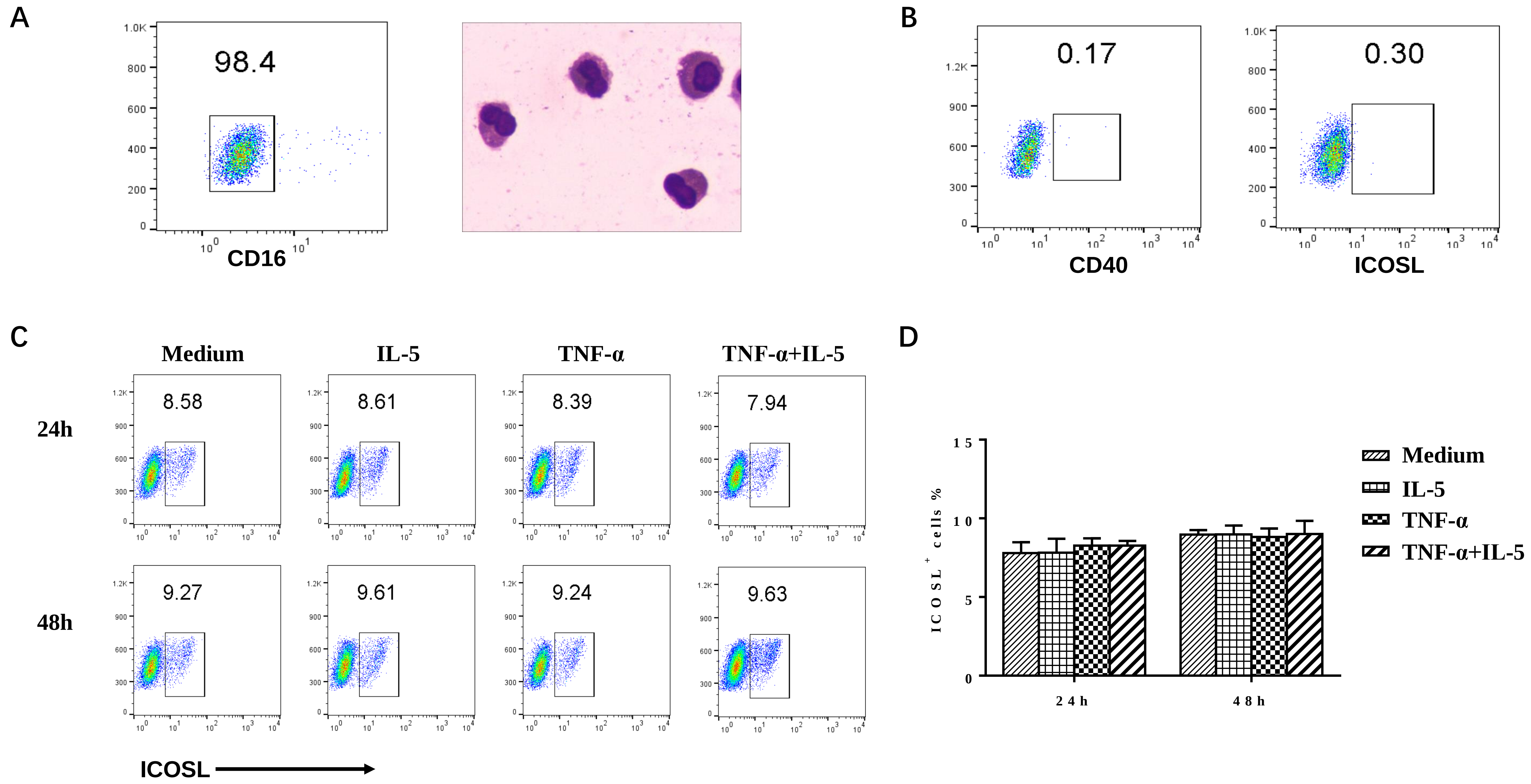

Supplementary figure 2

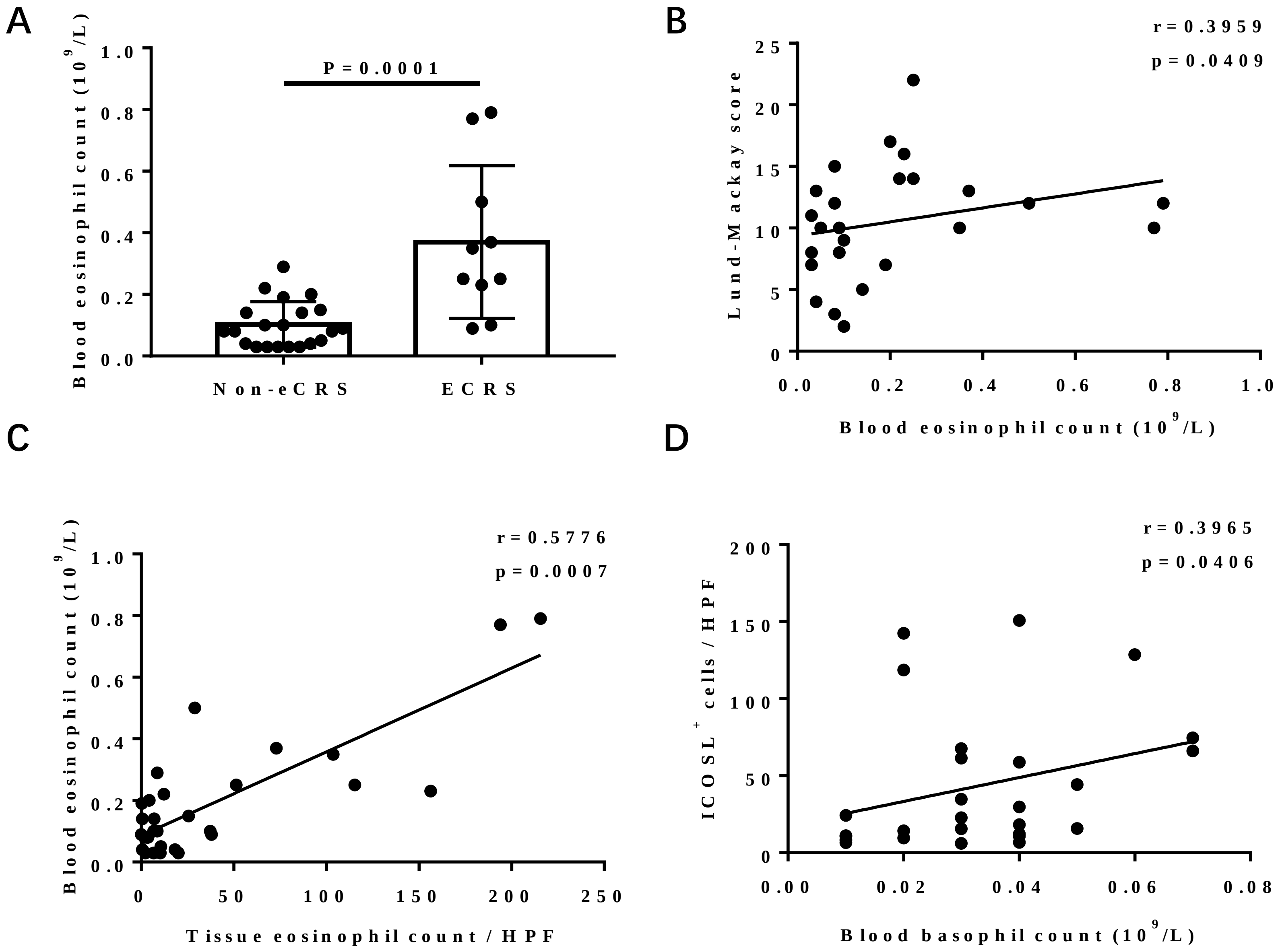

Supplementary figure 3
